# Empagliflozin improves vascular insulin sensitivity and muscle perfusion in persons with type 2 diabetes

**DOI:** 10.1152/ajpendo.00267.2023

**Published:** 2024-01-03

**Authors:** Linda A. Jahn, Lee M. Hartline, Thi Nguyen, Kevin Aylor, William B. Horton, Zhenqi Liu, Eugene J. Barrett

**Affiliations:** ^1^Division of Endocrinology, Department of Medicine, University of Virginia, School of Medicine, Charlottesville, Virginia, United States; ^2^Department of Pharmacology, University of Virginia, School of Medicine, Charlottesville, Virginia, United States

**Keywords:** blood pressure, endothelium, vascular insulin resistance

## Abstract

Sodium glucose cotransporter 2 inhibitors (SGLT2is) improved major adverse cardiovascular events (MACE), heart failure, and renal outcomes in large trials; however, a thorough understanding of the vascular physiological changes contributing to these responses is lacking. We hypothesized that SGLT2i therapy would diminish vascular insulin resistance and improve hemodynamic function, which could improve clinical outcomes. To test this, we treated 11 persons with type 2 diabetes for 12 wk with 10 mg/day empagliflozin and measured vascular stiffness, endothelial function, peripheral and central arterial pressures, skeletal and cardiac muscle perfusion, and vascular biomarkers before and at 120 min of a euglycemic hyperinsulinemic clamp at *weeks 0* and *12*. We found that before empagliflozin treatment, insulin infusion lowered peripheral and central aortic systolic pressure (*P* < 0.05) and muscle microvascular blood flow (*P* < 0.01), but showed no effect on other vascular measures. Following empagliflozin, insulin infusion improved endothelial function (*P* = 0.02), lowered peripheral and aortic systolic (each *P* < 0.01), diastolic (each *P* < 0.05), mean arterial (each *P* < 0.01), and pulse pressures (each *P* < 0.02), altered endothelial biomarker expression, and decreased radial artery forward and backward pressure amplitude (each *P* = 0.02). Empagliflozin also improved insulin-mediated skeletal and cardiac muscle microvascular perfusion (each *P* < 0.05). We conclude that empagliflozin enhances insulin’s vascular actions, which could contribute to the improved cardiorenal outcomes seen with SGLT2i therapy.

**NEW & NOTEWORTHY** The physiological underpinnings of the cardiovascular benefits of SGLT2 inhibitors remain uncertain. We tested whether empagliflozin mitigates vascular insulin resistance in patients with type 2 diabetes. Aortic and peripheral systolic, diastolic, mean and pulse pressures, endothelial function, vascular stiffness, and heart and muscle microvascular perfusion were measured before and during an insulin infusion at baseline and after 12 wk of empagliflozin. After empagliflozin, vascular responses to insulin improved dramatically.

## Graphical abstract

**Figure:**
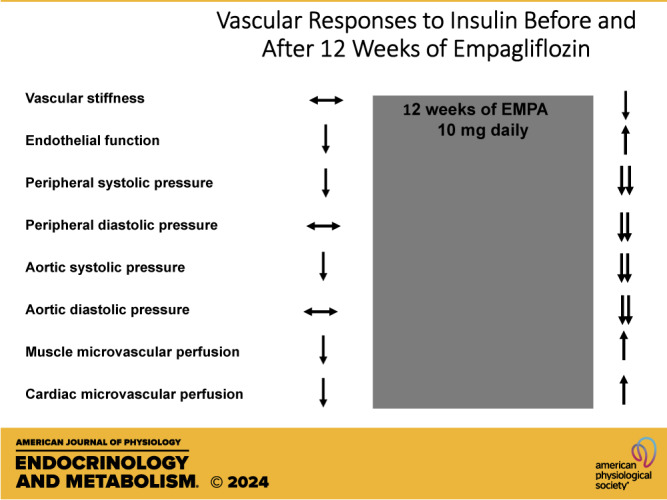

## INTRODUCTION

The sodium-glucose cotransporter-2 inhibitor (SGLT2i) class of pharmaceuticals dramatically improves important clinical outcomes in patients with type 2 diabetes (T2D) (1) and cardiovascular disease (2, 3) by uncertain mechanisms (4, 5). SGLT2i inhibits renal proximal tubule sodium-dependent glucose reabsorption, with resultant glycosuria, natriuresis, and diuresis. Secondary effects, derived directly from these target effects include a lowered fasting plasma glucose, modestly decreased resting systolic and diastolic blood pressures (6), and some weight loss. Other effects of SGLT2i treatment [e.g., shift in metabolic fuel usage toward fat oxidation (7), decreasing inflammatory biomarkers (8), and enhancing myocardial performance (9, 10)] have a more complex etiology and may be either secondary to the targeted SGLT2i responses or off-target actions of these drugs. Recent unbiased proteomic analysis found significant concentration changes of > 40 plasma proteins in participants with impaired glucose tolerance or type 2 diabetes treated with 25 mg of empagliflozin daily for 4 wk (11). Underscoring the pleiotropic spectrum of this response, these proteins included multiple representatives from diverse physiological domains including iron homeostasis, inflammation, fat metabolism, IGF-1 transport, and others.

Resistance to insulin-mediated glucose disposal is a core component of type 2 diabetes and is commonplace in obesity, metabolic syndrome, chronic renal disease (12), and heart failure(13). SGLT2i treatment improves systemic glucose metabolism (14) and muscle insulin sensitivity (15) in patients with T2D. In addition to its metabolic effects on glucose, protein, and lipid metabolism, insulin acts directly on vascular tissues and can enhance endothelial function (16), lower blood pressure (17), increase blood flow (18), decrease vascular stiffness (19), and enhance capillary recruitment in muscle (20), heart (21), and adipose (22) tissues in healthy adults. We and others have noted that insulin resistance, as seen with obesity, metabolic syndrome, PCOS, and type 2 diabetes, extends to insulin’s vascular actions (18, 23–26).

To our knowledge, direct measurements of the impact of GLT2i treatment on vascular insulin action are currently unavailable in humans. We therefore examined the impact of 12 wk of treatment with 10 mg of empagliflozin daily on micro- and macrovascular insulin sensitivity in persons with well-controlled type 2 diabetes. Although specifically studying people with type 2 diabetes, we considered that if empagliflozin treatment enhanced vascular insulin sensitivity, such an action might also at least partially explain the beneficial cardiovascular and renal effects of this drug class in nondiabetic patients with cardiovascular disease (CVD), heart failure (13), or chronic renal disease (27), each condition itself being associated with insulin resistance.

## METHODS

### Human Participants

Participants with type 2 diabetes were recruited through newspaper ads, posters, and letters mailed to patients followed at University of Virginia outpatient clinics. Respondents were contacted by telephone to review inclusion/exclusion criteria. Participants taking insulin, SGLT2i, GLP-1 receptor agonists, having a body mass index (BMI) >39 kg/m^2^, or with active cardiac, renal, hepatic or central nervous system illnesses were excluded. Subsequently, each participant completed a written informed consent and underwent a screening visit that included a medical history and physical exam, and measurements of serum electrolytes, liver, and renal function, a complete blood count, fasting glucose, C-peptide, hemoglobin A1c, a lipid panel, and a pregnancy test (if applicable). Forty-seven participants responded to letter or advertisement solicitations. Of these respondents, 25 participants did not meet prescreening criteria. Twenty-two participants ultimately passed prescreening and were invited for a dedicated, in-person screening examination. Of those 22, six failed the screening, one dropped out before starting the study, and four had IV line failures during their first admission and subsequently withdrew. This resulted in 11 participants completing the study. Two insulin clamp studies were performed on these 11 participants with at least a 1-year history of type 2 diabetes based on the American Diabetes Association criteria. Seven participants were on stable doses of antihypertensive medications at the time of recruitment and seven were on statins. These were unchanged during the study. The study protocol was approved by the University of Virginia Institutional Review Board. All infusion studies were performed in the University of Virginia Clinical Research Unit (CRU).

This study was conducted under NCT# 04203927.

#### Experimental protocol.

**Figure 1.**
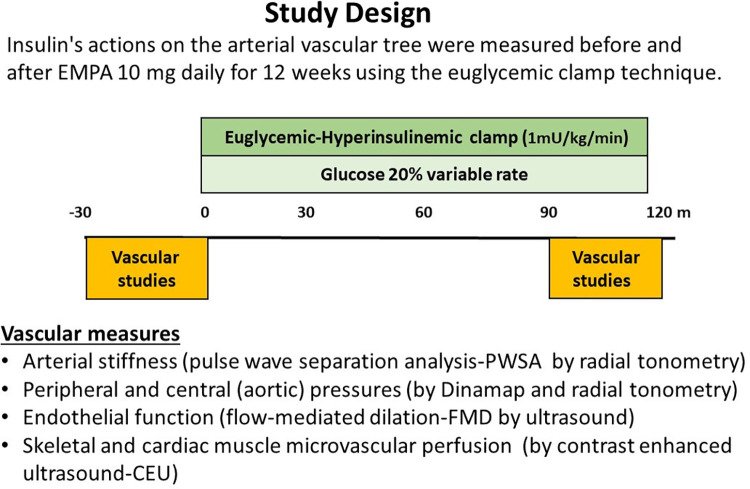
Study design for testing vascular insulin sensitivity. Baseline measures of plasma insulin and vascular biomarkers along with measures of endothelial function (FMD), peripheral and aortic pressures, vascular stiffness (AI, PWSA), and skeletal and myocardial microvascular perfusion (CEU) were obtained between *minutes −30* and *0*. At *time 0*, a primed-continuous insulin infusion was begun and continued for 120 min. Plasma glucose was measured every 5 min for 120 min and the infusion rate of a 20% dextrose solution adjusted to maintain euglycemia. All vascular biomarker and vascular function measurements were repeated between 90 and 120 min. Each participant was studied on two occasions, before and at the end of 12 wk of empagliflozin treatment. AI, augmentation index; CEU, contrast-enhanced ultrasound; FMD, flow-mediated dilation; PWSA, pulse wave separation analysis.

Figure 1 illustrates the study design for measuring vascular and metabolic insulin action. Participants were admitted to the CRU at 7 AM after an overnight fast both at *week 0* (before treatment) and at the end of the 12-wk empagliflozin treatment period. Indices of vascular function were measured at baseline and again during the final 30 min of a 120-min, 1 mU/kg/min euglycemic insulin clamp. An intravenous catheter was placed in the right antecubital vein for infusion of glucose, insulin, and ultrasound contrast agent (Definity microbubbles Lantheus Medical Imaging, N. Billerica, MA) and a second catheter was placed at the right wrist for blood sampling. Fasting plasma glucose was measured and if outside a target range of 100–140 mg/dL, a slow infusion of either glucose (1 mg/kg/min) or insulin (0.15 mU/kg/min) was begun and continued until plasma glucose was within the desired target range. We then obtained baseline (*time 0*) measurements of metabolic parameters, endothelial biomarkers, and vascular functions. These included plasma glucose, insulin, vascular cell adhesion molecule 1 (VCAM1), intercellular adhesion molecule 1 (ICAM1), platelet endothelial cell adhesion molecule 1 (PECAM1), endothelin 1 (ET1), e-Selectin, and S100A8/9. We measured vascular stiffness by carotid-femoral pulse wave velocity (cfPWV), and augmentation index (AI) with pulse wave separation analysis (PWSA). Peripheral blood pressure was measured at the brachial artery while central aortic blood pressure and pulse pressures were measured by radial artery tonometry. Brachial artery flow-mediated dilation (FMD) was measured by B-mode ultrasound while measures of skeletal and cardiac muscle microvascular blood volume, microvascular flow velocity, and microvascular blood flow were obtained using contrast-enhanced ultrasound (CEU). After these measurements were completed, a 1 mU/kg/min hyperinsulinemic-euglycemic clamp was begun and endothelial plasma markers and vascular measurements were repeated between 90 and 120 min of the clamp. Glucose was measured every 5 min and insulin every 30 min during the clamp.

Between *weeks 0* and *12*, participants continued all of their usual medications in addition to 10 mg of empagliflozin daily in the morning. Serum electrolytes, BUN, and creatinine were obtained at *weeks 0, 4, 8*, and *12*.

### Measures of Conduit Vessel Function

Vascular stiffness was assessed by measuring both PWSA and carotid-femoral PWV using aplanation tonometry (SphygmoCor System, AtCor Medical, Itasca, IL). For PWSA, we used the radial artery and the measured augmentation index (AI, corrected to a heart rate of 75 beats/min), the forward (Pf) and backward (Pb) pulse wave amplitude as well as reflection magnitude (RM = Pb/Pf) (28). Measurement of peripheral mean arterial (P-MAP) pressures and pulse pressure (P-PP) as well as calculation using a validated transform of aortic systolic (A-Sys), diastolic (A-Dia), mean aortic (A-MAP), and aortic pulse pressure (A-PP) were obtained using the SphygmoCor tonometer.

### Endothelial Function

FMD was measured using an Epiq 7 ultrasound system (Phillips Medical). The brachial artery was imaged ∼5 cm proximal to the antecubital crease using B-mode ultrasound. Baseline brachial artery diameter was measured and a forearm blood pressure cuff was inflated to 250 mmHg for 5 min and then deflated. Diameter was obtained every 10 s from 30–180 s after cuff deflation. Analysis for vessel diameter was done offline using Brachial Analyzer (Medical Imaging Applications, LLC, Coralville, IA).

### Microvascular Perfusion by CEU

Cardiac (i.e., interventricular septum) and forearm flexor muscle microvascular perfusion were assessed with an Epiq 7 ultrasound system during steady-state infusion of Definity microbubbles (Lantheus Medical Imaging; North Billerica, MA) using low mechanical index (MI = 0.10) continuous imaging for 20 s (myocardium) or 30 s (forearm muscle) at a frame rate of 15 s^−1^ with a flash at 0.88 MI to initiate a replenishment curve (29). Four forearm and four myocardial replenishment curves were acquired immediately before starting and at the end of the insulin clamp study (Fig. 1). Replenishment curves were analyzed using Q-Lab software (Philips Research; Cambridge, MA) yielding measures of microvascular blood volume (MBV, in video intensity units) and microvascular flow velocity (MFV, in s^−1^). Their product provides a measure of microvascular blood flow (MBF, in VI × s^−1^).

### Euglycemic Hyperinsulinemic Clamp

After obtaining baseline plasma samples and vascular measurements, a 2 mU/kg/min insulin infusion was begun and decreased to 1 mU/kg/min after 10 min and maintained until 120 min. During the insulin clamp, to avoid the confounding systemic vasodilation due to limb heating (30, 31), we did not use a “heated hand” to arterialize venous blood, as previously described (32).

### Continuous Glucose Monitoring

All participants were provided a continuous, subcutaneous glucose monitor (Abbott Freestyle Libre, kindly provided by Abbott Diabetes Care; Alameda, CA) and instructed in its use. Masked CGM data were downloaded every 14 days and measures of glucose control (e.g., mean glucose, time in range, etc.) and variability (e.g., standard deviation and coefficient of variation) were recorded and the sensor changed.

### Biochemical Analyses

Serum electrolytes, creatinine, liver function, HbA1c, complete blood counts, lipid profiles, and pregnancy tests were assayed at the University of Virginia Clinical Chemistry Laboratory. Plasma glucose was measured using a YSI glucose analyzer (Yellow Spring Instruments, Inc.; Yellow Springs, OH). Plasma insulin was determined by ELISA (Alpco Corp., Salem, NH) as were the plasma concentrations of VCAM1, ICAM1, PECAM1, e-Selectin, S100A8/9, and endothelin1 (ET1) using antibodies from R&D Systems (Minneapolis, MN).

### Statistics

The primary endpoint for microvascular insulin responsiveness was the change in forearm muscle MBV from baseline to end-of-clamp. The primary endpoint for macrovascular insulin responsiveness was arterial stiffness (Pf, Pb, and RM). Central and peripheral blood pressure changes and FMD changes were secondary endpoints. For assessing metabolic insulin sensitivity, we used the glucose infusion rate (GIR) needed to maintain euglycemia during the clamp. Data are presented as the mean ± standard error of the mean (SE). Comparisons were made between treatment (pre- vs. postempagliflozin) and between before (*time 0*) and after (at 90–120 min) of the insulin clamp using paired Student’s *t* test, two-way ANOVA, and Pearson’s correlation as necessary. All statistical analyses were conducted with GraphPad Prism Software (Dotmatics, Boston, MA). Statistical significance was defined as *P* ≤ 0.05 for primary and secondary endpoints.

## RESULTS

Clinical and phenotypic characteristics of the study cohort at *weeks 0* and *12* are given in Table 1. Participants at entry were middle-aged nonsmokers with reasonably well-controlled type 2 diabetes. Participants were not taking any over-the-counter medications or supplements known to affect vascular function (e.g., fish oil, vitamins E or C, or aspirin). Over 12 wk, there was a modest but significant decline in BMI and fasting glucose along with a downtrend of systolic and diastolic BP and triglycerides.

**Table 1.** Clinical and biochemical characteristics before and after 12 wk of empagliflozin treatment

	*Week 0*	*Week 12*	*P* Value
Sex, M/F	6M/5F	6M/5F	ND
Age, yr	55 ± 2	55 ± 2	ND
BMI, kg/m^2^	33.8 ± 1.4	32.8 ± 1.4	<0.01
Fasting glucose, mg/dL	128 ± 11	110 ± 7	0.02
Systolic BP, mmHg	127 ± 6	125 ± 2	0.75
Diastolic BP, mmHg	75 ± 3	72 ± 4	0.09
Total cholesterol, mg/dL	154 ± 13	142 ± 14	0.13
LDL-cholesterol, mg/dL	89 ± 12	81 ± 12	0.20
HDL-cholesterol, mg/dL	39 ± 2	37 ± 3	0.14
Triglycerides, mg/dL	161 ± 21	146 ± 21	0.50
Hemoglobin A1c, %	7.01 ± 0.4	6.7 ± 0.3	0.10
C-peptide, ng/mL	5 ± 0.9	ND	ND
Creatinine, mg/dL	0.8 ± 0.2	0.79 ± 0.3	0.88
Glucose infusion rate, mg/kg/min	2.43 ± 0.4	2.76 ± 0.4	0.07

Data reported as means ± SE. *P* value for paired *t* tests, *n* = 11 subjects. BMI, body mass index.

CGM data were available for 10 of 11 participants and indicated good glycemic control throughout the 12 wk of study with a mean glucose of 130 ± 2 mg/dL, an estimated A1c of 6.4 ± 0.05%, and an average time in glucose target range of 80 ± 1%. There were no episodes of ketosis or severe hypoglycemia during the 12-wk study period.

### Metabolic Insulin Sensitivity

At *week 0*, after the overnight fast and before beginning the insulin clamp, the plasma glucose concentration averaged 120 ± 6 mg/dL and the plasma insulin concentration 23 ± 6 mIU/L. At 120 min of euglycemic-hyperinsulinemia, the steady-state glucose infusion rate averaged 2.5 ± 0.5 mg/kg/min and the steady-state plasma insulin concentration 126 ± 16 mIU/L. After 12 wk of empagliflozin treatment, the fasting glucose (108 ± 5 mg/dL) was significantly lower (*P* < 0.01), fasting plasma insulin trended lower (18 ± 5 mU/L, *P* < 0.06), and average GIR trended higher 2.8 ± 0.4 mg/kg/min (*P* = 0.07) with a mean steady-state plasma glucose and insulin concentrations of 105 ± 3 mg/dL and 128 ± 11 mIU/L, respectively. As neither hepatic glucose production nor urine glucose losses were measured (each of which increases with SGLT2i treatment and which has somewhat offsetting effects on estimates of whole body or muscle glucose uptake), the estimates of metabolic insulin resistance derived from the clamp studies are approximate. However, given the very low GIR values during both clamps compared with the GIR typical of middle-age, lean healthy controls receiving the identical insulin infusion [∼6 mg/kg/min, (32, 33)], it was evident that these participants with type 2 diabetes are metabolically insulin resistant both before and after empagliflozin treatment.

### Endothelial Function

Brachial artery FMD measurements were available in 10 of 11 participants (one participant required intravenous access in both arms that prevented FMD measurements). At *week 0*, FMD averaged 7.6 ± 1.2% before insulin and 6.0 ± 0.9% after insulin (Table 2). That downward trend was not statistically significant. At *week 12* of empagliflozin, before giving insulin, the FMD was 7.4 ± 1.1%. This was not different from values seen before empagliflozin treatment. After insulin, FMD was 8.7 ± 1.5%, which was significantly higher than the postinsulin FMD seen before empagliflozin (*P* = 0.02).

**Table 2.** Effect of insulin on peripheral and central blood pressures before and after 12 wk of empagliflozin

	*Week* *0*			*Week* *12*			
	0 min	120 min	*P* value	0 min	120 min	*P* value	*P* value Insulin ± Empa
Peripheral pressures, mmHg							
P SBP	133 ± 6	125 ± 5	<0.04	126 ± 6	109 ± 5	<0.002	<0.001
P DSP	72 ± 2	72 ± 2	ns	74 ± 4	66 ± 3	<0.01	<0.05
P MAP	96 ± 4	90 ± 2	ns	91 ± 3	80 ± 3	<0.01	<0.001
P PP	58 ± 6	54 ± 5	ns	53 ± 6	43 ± 3	<0.05	<0.04
Central aortic pressures, mmHg							
A SBP	122 ± 6	114 ± 4	<0.03	115 ± 5	98 ± 4	<0.001	<0.001
A DSP	77 ± 4	72 ± 2	ns	74 ± 3	67 ± 3	<0.05	ns
A MAP	92 ± 4	90 ± 2	ns	89 ± 3	77 ± 3	<0.01	<0.002
A PP	44 ± 5	42 ± 6	ns	43 ± 4	30 ± 2	<0.05	<0.04

Data presented as means ± SE. *P* values refer to paired *t* tests before and after insulin at *weeks 0* and *12*, and after insulin before and after empagliflozin, *n* = 10 persons. DSP, diastolic blood pressure; MAP, mean arterial pressure; PP, pulse pressure; SBP, systolic blood pressure.

### Plasma Concentrations of Endothelial Cell Marker Proteins

**Figure 2.**
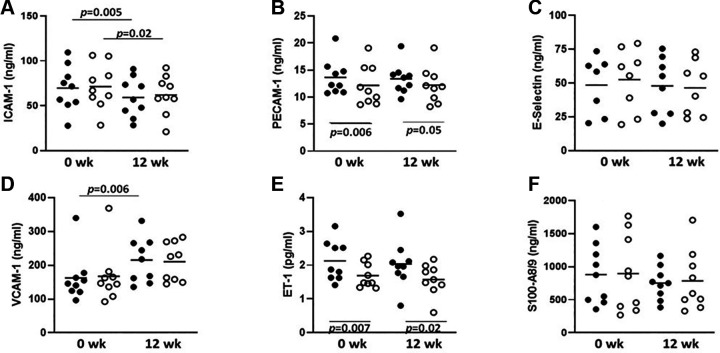
Plasma concentrations of endothelial cell biomarkers obtained before (filled circles) and after (open circles) 120 min of euglycemic hyperinsulinemia, at *weeks 0* and *12* of empagliflozin treatment. *A*: ICAM-1. *B*: PECAM-1. *C*: E-selectin. *D*: VCAM-1. *E*: ET-1. *F*: S100-A8/9. *P* values above data points indicate differences before and after empagliflozin, whereas *P* values below data points indicate comparisons before and after insulin. All measures are mean of duplicates, *n* = 9 persons. ET-1, endothelin 1; ICAM-1, intercellular adhesion molecule 1; PECAM-1, platelet endothelial cell adhesion molecule 1; VCAM-1, vascular cell adhesion molecule 1.

We measured plasma concentrations of several endothelial cell-derived markers to assess whether empagliflozin affected either baseline secretion of these markers or was affected by insulin acting on the endothelium. We observed that ICAM1 concentrations declined significantly in response to SGLT2i treatment (Fig. 2), but there was no effect of insulin on either the baseline or 12-wk plasma ICAM1 concentration. Conversely, VCAM1 significantly increased between *weeks 0* and *12*. VCAM1 concentration was also unresponsive to insulin at either *week 0* or *12*. Empagliflozin treatment did not affect plasma levels of either ET1 or PECAM1 but both declined significantly in response to the insulin infusion before and after empagliflozin. E-selectin and S100A8/9 concentrations did not change with either empagliflozin treatment or insulin infusion (Fig. 2).

### Peripheral Arterial and Central Aortic Pressures

**Figure 3.**
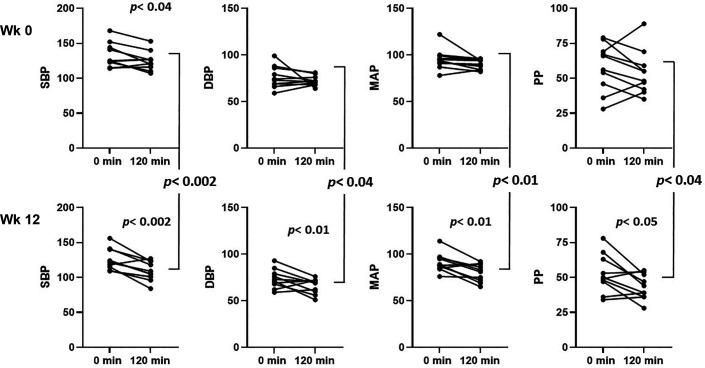
Peripheral systolic, diastolic, mean arterial, and pulse pressures (mmHg) measured before (0 min) and at 120 min of euglycemic hyperinsulinemia both before (*top*) and at 12 wk of empagliflozin treatment (*bottom*). *P* values for the effect of insulin are below symbols, *P* values for effect of empagliflozin are between the *top* and *bottom* panels; *n* = 9 persons.

These pressures were obtained in 10 of 11 participants (frequent ectopy prevented their collection in one participant). Before empagliflozin treatment, peripheral systolic arterial pressure (P-Sys) averaged 133 ± 6 mmHg, peripheral diastolic pressure (P-Dia) 76 ± 4 mmHg, peripheral mean arterial pressure (P-MAP) 96 ± 4 mmHg, and peripheral pulse pressure 58 ± 6 mmHg. Insulin infusion for 2 h lowered P-Sys (*P* < 0.04) but did not significantly affect other peripheral pressures (Table 3 and Fig. 3). After empagliflozin treatment, these peripheral pressures before giving insulin averaged modestly lower than *week 0* (126 ± 5, 74 ± 3, 91 ± 3, and 53 ± 5 mmHg, respectively); however, these changes were not significantly different than at *week 0*. By contrast, addition of insulin for 2 h at 12 wk of empagliflozin treatment significantly lowered P-Sys, P-Dia, P-MAP, and P-PP to 109 ± 5, 66 ± 3, 80 ± 3, and 43 ± 3 mmHg, respectively (Table 3 and Fig. 3). These latter more pronounced BP declines occurred despite preinsulin pressures that were already lower than at *week 0* and resulted in pressures that were significantly lower than the postinsulin pressures at *week 0* (Fig. 3).

**Table 3.** Effect of insulin on measures of endothelial function (FMD) and vascular stiffness (PWSA) before and after 12 wk of empagliflozin

	*Week 0*			*Week 12*			
Insulin	0 min	120 min	*P* value	0 min	120 min	*P* value	*P* value insulin ± Empa
Flow-mediated dilation	7.8 ± 1.1	6.2 ± 0.9	0.15	7.5 ± 1.1	8.8 ± 1.4	0.31	<0.02
Forward pressure wave (pf) mmHg	28.3 ± 4.0	29.2 ± 3.6	0.62	28.6 ± 2.4	22.8 ± 2.4	<0.02	0.09
Backward pressure wave (pb) mmHg	21.5 ± 2.8	20.2 ± 3.2	0.55	19.1 ± 2.3	13.2 ± 1.0	<0.02	0.09
Reflection maximum (RM) %	70.4 ± 6.7	68.4 ± 5.8	0.61	60.8 ± 6.4	58.8 ± 5.0	0.75	0.98

Data presented as means ± SE. FMD, flow-mediated dilation; PWSA, pulse wave separation analysis.

**Figure 4.**
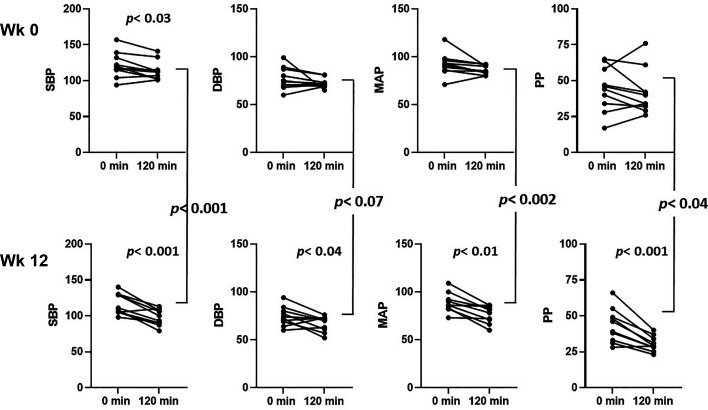
Aortic systolic, diastolic, mean arterial, and pulse pressures (mmHg) measured before (0 min) and at 120 min of euglycemic hyperinsulinemia both before (*top*) and at 12 wk of empagliflozin treatment (*bottom*). *P* values for the effect of insulin are below symbols, *P* values for effect of empagliflozin are between the *top* and *bottom* panels. *P* values are by paired *t* tests, *n* = 10 persons.

As with peripheral arterial pressures, aortic systolic (A-Sys) and diastolic (A-Dia) pressures, as well as aortic mean arterial pressure (A-MAP) and aortic pulse pressure (A-PP) declined modestly (significant only for A-Sys) with insulin infusion before empagliflozin treatment (Table 3 and Fig. 4). However, after 12 wk of empagliflozin, A-Sys and A-Dia pressures as well as A-MAP and A-PP all declined significantly during insulin infusion (see Table 3 and Fig. 4). Furthermore, A-Sys, A-Dia pressures, A-MAP, and A-PP were each significantly lower at *week 12* during insulin infusion than during insulin infusion at *week 0*. Particularly striking was the greater than twofold insulin-induced declines of P-Sys and A-Sys pressures as well as P-PP and A-PPs after empagliflozin treatment (Table 3) when compared with *week 0*. We noted that these larger insulin-induced decrements occurred despite lower preinsulin pressures in both the central and peripheral arterial circulation.

### Arterial Stiffness (AI, PWSA, and cfPWV)

Table 2 shows values for forward wave pressure (Pf), backward wave pressure (Pb), and reflection maximum before and at the end of the euglycemic insulin clamp at *weeks 0* and *12*. The AI was 19.6 ± 3.3% before insulin at *week 0* and 17.4 ± 2.5% at *week 12* and did not change in response to insulin infusion at either *week 0* or *12* before or with empagliflozin treatment. At *week 0*, insulin infusion had no effect on the observed forward and backward arterial waveforms. At *week 12*, there was no change in the preinsulin pressure wave profile compared with the *week 0* baseline values. However, insulin infusion at *week 12* significantly decreased the height of both the forward and backward pressure waves (*P* = 0.02, each). In addition, both the forward and backward pressure waves after insulin trended lower at *week 12* compared with the *week 0* postinsulin values (*P* = 0.08 and *P* = 0.05, respectively). cfPWV measurements could only be obtained in 5/11 participants secondary to poor consistency of carotid or femoral waveforms obtained in more than half of the patients and are not reported here.

### Skeletal Muscle Microvasculature

**Figure 5.**
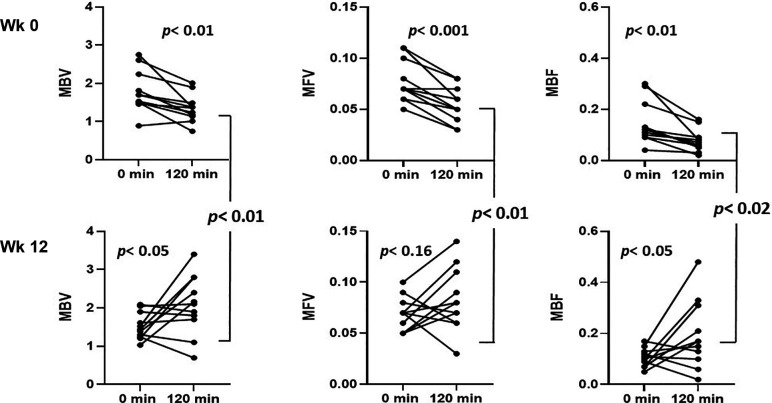
Effect of insulin infusion on indices of forearm muscle microvascular perfusion including MBV (steady-state CEU video intensity in arbitrary units), microvascular flow velocity (MFV in s^−1^) and their product MBF (AU × s^−1^) at *week 0* (*top*) and after 12 wk of empagliflozin treatment (*bottom*). CEU, contrast-enhanced ultrasound; MBF, microvascular blood flow; MBV, microvascular blood volume; MFV, microvascular flow velocity. *P* values by paired *t* tests for 11 persons.

Before empagliflozin treatment, insulin infusion decreased skeletal muscle MBV by 22% (*P* < 0.01), MFV by 31% (*P* < 0.01), and MBF by 45% (*P* < 0.01) (Fig. 5). MBV declined in 10/11, MFV in 10/11, and MBF in 11/11 participants, which is consistent with insulin inducing a vasoconstrictive response in these insulin-resistant subjects. After treatment with empagliflozin, the baseline (0 min) measures of skeletal muscle MBV, MFV, and MBF obtained before insulin did not differ from those obtained on *week 0* before empagliflozin. However, now insulin increased MBV, MFV, and MBF in 7/11 participants for each and the average MBV, MFV, and MBF increased by 43%, 27%, and 104%, respectively. These percent increments were greater (*P* ≤ 0.01 for each) compared with the *week 0* response to insulin. The postinsulin MBV, MFV, and MBF at *week 12* were greater (*P* ≤ 0.02, for each) than respective postinsulin, pre-empagliflozin values (Fig. 5).

### Myocardial Microvasculature

Before and after empagliflozin data were only available for 8 of 11 participants as one participant had excessive ectopy, in another we were unable to achieve adequate vascular access, and the third had a poor echocardiogram window that precluded appropriate cardiac imaging. Before empagliflozin treatment, in response to insulin, there were no significant changes in heart MBV, MFV, or MBF. After empagliflozin treatment, there was no difference in baseline MBV, MFV, or MBF before insulin infusion. After insulin, MBV trended higher (*P* = 0.067) and both MFV (*P* = 0.049) and MBF (*P* = 0.048) were significantly greater than their respective *week 0* postinsulin values.

### Results Summary

There were no differences before and after empagliflozin in the baseline (preinsulin infusion) measures of endothelial function (FMD), vascular stiffness (Pf and Pb) and skeletal and cardiac muscle microvascular blood volume (MBV), microvascular flow velocity (MFV), and microvascular blood flow (MBF). For peripheral and central pressures, insulin provoked a vasodilatory response both before and after empagliflozin treatment, the extent of vasodilation was simply greater after 12 wk of empagliflozin despite lower preinsulin pressures.

## DISCUSSION

We provide here novel evidence for a potent action of empagliflozin to increase vascular insulin sensitivity in persons with type 2 diabetes and insulin resistance. The effect of empagliflozin treatment on insulin’s vascular actions in this exploratory study was observed across a broad panel of macro- and microvascular functions and measures of vascular reactivity. These included measures of endothelial function and endothelial adhesion protein release into plasma, indices of vascular stiffness, central and peripheral blood pressures, and measures of microvascular perfusion in both skeletal and cardiac muscle. We considered that weight loss or improved glycemia might be contributors or intermediates in the improved vascular function after insulin. However, we found no significant correlation between changes in BMI or A1c and changed responses to insulin of endothelial function (change of FMD), vascular stiffness (forward or backward pressure wave amplitude), or skeletal muscle perfusion when compared with before and after empagliflozin treatment. We recognize that the sensitivity of this correlation analysis is limited due to both the small number of subjects studied and the small changes seen in BMI and A1c. Whether the changes in vascular insulin sensitivity we observed (individually or in aggregate) contribute meaningfully to the observed beneficial MACE, heart failure, or functional renal preservation seen in long-term, multicenter clinical trials cannot be adjudicated from the current small, 12-wk trial. Rather, the current data strongly support a novel hypothesis that empagliflozin (and possibly SGLT2is in general) has a striking and previously unappreciated ability to enhance or restore insulin’s vascular actions.

We considered it important to test for an interaction between empagliflozin and insulin’s actions on vascular function, given empagliflozin’s strong impact on heart failure, CVD, and renal function outcomes and given the association of each of these pathologies with metabolic insulin resistance. As noted previously, to our knowledge, there have been no reports testing the impact of empagliflozin treatment on vascular insulin sensitivity. Available data indicate that insulin’s vascular actions on endothelial cells result from the stimulation of two kinase pathways downstream of its receptor, one activating PI-3 kinase, Akt, and eNOS resulting in enhanced nitric oxide production, the other activating ERK½ signaling and increasing endothelin1 synthesis (34). In health, insulin infusion promotes vasodilation, consistent with a dominant effect to promote NO signaling. Insulin induced vasodilation is diminished with metabolic syndrome, type 1 or type 2 diabetes, or obesity. Whether empagliflozin treatment in the current study is impacting either or both of these endothelial cell insulin-directed kinase cascades or acting through other mechanisms is unknown. Indeed, whether the vascular effects of empagliflozin we observed are mediated directly by modulating insulin’s actions on endothelium, on arterial smooth muscle cells, or both or result from some more indirect path is uncertain. More invasive preclinical studies with measurement of insulin responsiveness of vascular cell populations with or without empagliflozin treatment are required. Such studies could also help clarify whether empagliflozin’s vascular effects are direct or a consequence of an altered metabolic milieu provoked by renal SGLT2 inhibition. Recent data suggest that while endothelial cells express SGLT2, empagliflozin does not significantly depress glucose uptake by these cells (35). Despite this, it improved endothelial cell chemotaxis and reduced cell reactive oxygen species (ROS) production and inflammation in response to hyperglycemia, seemingly by pathways independent of glucose transport.

We noted that endothelial function (FMD) and skeletal and cardiac microvascular function trended downward during insulin infusion before empagliflozin treatment while both trended higher after empagliflozin. We considered that this may reflect a shift in the balance of insulin acting on phosphotidyl-inositol-3-kinase versus MEK ½ signaling leading to increased nitic oxide or decreased endothelin1 secretion (or both) with insulin after empagliflozin treatment. However, we also note that the negative vascular responses to insulin did not extend to measurements of systolic blood pressure (which declined with insulin in both the peripheral and central circulation before and after empagliflozin treatment). Likewise, the forward and backward components of the pressure wave (Pf and Pb) did not change with insulin before empagliflozin but declined during the clamp after empagliflozin treatment. The effect of empagliflozin on peripheral and central blood pressure responses to physiological increments of plasma insulin in the current study were striking. Chilton et al. (36) performed post hoc analyses of peripheral blood pressure data from several multicenter clinical trials of empagliflozin treatment in individuals with diabetes and cardiovascular disease. They noted that empagliflozin reduced systolic and diastolic blood pressures by ∼4 and ∼2 mmHg, respectively, without increasing heart rate. The decline in double product (HR × SBP) signaled a decreased workload to the heart. They also observed (from 24 h blood pressure recordings) a nonsignificant decline of the ambulatory arterial stiffness index. These changes, while statistically significant, did not appear to be of a magnitude sufficient to account for the striking CVD and renal benefits observed. In the current study, 12 wk of empagliflozin lowered both systolic and diastolic pressures before insulin, but these changes were not statistically significant. At *week 0*, insulin infusion significantly lowered peripheral and central systolic pressures (Table 3). At *week 12*, insulin was significantly more effective in lowering peripheral systolic and diastolic pressures, as well as P-MAP and P-PP (Table 3 and Fig. 3) than at *week 0*. Insulin was also significantly more effective at lowering central aortic pressures after empagliflozin treatment (Fig. 4). This would be expected to decrease heart workload, as well as perfusion pressure in the renal, coronary, and cerebral circulation, which might diminish vascular injury in each circulation.

The improved peripheral and central pressure measurements suggest an effect of empagliflozin to augment insulin-mediated relaxation of resistance arterioles. In considering the more dramatic impact of insulin infusion on aortic versus peripheral pressures after empagliflozin, it is worth recalling the greater differential impact of nitroglycerin on aortic versus peripheral pressures (37, 38) as well as the aortic BP reduction synergy seen between nitroglycerin and sildenafil (39). Nitroglycerin and sildenafil each augment cGMP activity in vascular smooth muscle, mimicking the vasodilating action of endogenous endothelial-derived nitric oxide. Thus, a direct action of empagliflozin on vascular smooth muscle might account for the augmented BP lowering action of insulin in the current study.

Insulin, acting via PI-3-kinase, phosphorylates and activates endothelial nitric oxide synthase (eNOS), and we showed here that nitric oxide (NO)-dependent FMD was enhanced after insulin and empagliflozin exposure. An effect of empagliflozin to enhance eNOS sensitivity to insulin could explain the central and peripheral BP lowering and also explain the insulin-induced improvement of microvascular perfusion noted in both heart and skeletal muscle. We note that in skeletal muscle of healthy young adults, insulin has a consistent, vasodilatory action to enhance microvascular perfusion (24). In contrast, in insulin-resistant individuals, the responses to insulin infusion are heterogenous with vasorelaxation, vasoconstriction (33, 40), or no response observed. At *week 0* of the current study, all 11 participants had a vasoconstrictive MBF response to insulin in skeletal muscle. Conversely, in response to insulin at *week 12*, microvascular blood flow rose in seven participants and declined in four participants. The flow increase occurred despite the decline in P-Sys, indicating a decline in vascular resistance.

Treatment with empagliflozin also unmasked an action of insulin to decrease forward and backward wave peak magnitude. Such an action is consistent with an effect to lessen vascular stiffness. However, the acuity of the changes seen with insulin suggest any changes in stiffness are likely secondary to smooth muscle relaxation in the walls of conduit arteries. Interestingly, augmentation index (AI; another measure of vascular stiffness) did not change significantly after insulin at either 0 or 12 wk. As AI is a ratio of the augmentation wave height to the pulse pressure and the latter fell significantly, AI may not reflect a true measure of arterial stiffness in this circumstance.

We also tested a small set of biomarkers associated with endothelial cell function to assess whether they were affected by either empagliflozin, insulin, or a combination of the two. Interestingly, we observed significant insulin-induced declines of plasma concentrations of endothelin-1 and PECAM1. To our knowledge, this effect on PECAM1 has not been reported. We also observed significantly lower plasma concentrations of ICAM 1 along with raised plasma VCAM1 concentrations in response to empagliflozin treatment. There was no evidence for synergy between the effects of insulin and empagliflozin on any of the markers tested. The ICAM1 decline may signal lessened endothelial inflammation, a possibility that warrants dedicated investigation in future studies.

We recognize a number of significant limitations to the current study. These include *1*) we studied a limited number of participants with well-controlled type 2 diabetes without known microvascular complications; *2*) there was no placebo control group; *3*) the study lasted only 12 wk, hence, we cannot speculate on whether the observed effects endure; *4*) the insulin clamp, while extremely useful for testing insulin sensitivity, has no physiological parallel.

### Conclusions

In the current study, we identified marked and wide-ranging changes in vascular insulin sensitivity after 12 wk of empagliflozin therapy. We found these changes particularly surprising given that the metabolic insulin-sensitizing effects of SGLT2i therapy both in the current and other studies (14, 15) are modest. We were similarly surprised that we were unable to find previous reports of SGLT2I specifically affecting vascular insulin action. Given the new evidence for the significant beneficial effects of empagliflozin together with insulin on endothelial function, peripheral and central blood pressure, vascular stiffness, and microvascular perfusion of both heart and skeletal muscle, it will be important to assess the role of this interaction in other clinically relevant settings (e.g., type 2 diabetes patients with progressive renal insufficiency, heart failure, and/or ischemic heart disease with or without type 2 DM). Equally important will be sorting out the mechanism(s) for the interactions we have observed.

## ETHICAL APPROVALS

The experimental protocol was approved by the University of Virginia Institutional Review Board for Biomedical Studies.

## DATA AVAILABILITY

The datasets used and/or analyzed during the current study are available from the corresponding author on reasonable request.

## GRANTS

This work was supported by a research grant from the NIH National Heart, Lung, and Blood Institute R01-142240 to E.J.B.

## DISCLOSURES

No conflicts of interest, financial or otherwise, are declared by the authors.

## AUTHOR CONTRIBUTIONS

L.A.J., L.M.H., Z.L., and E.J.B. conceived and designed research; L.A.J., L.M.H., T.N., K.A., and W.B.H. performed experiments; L.A.J., T.N., K.A., and W.B.H. analyzed data; L.A.J., T.N., and Z.L. interpreted results of experiments; L.A.J. and T.N. prepared figures; L.A.J., L.M.H., W.B.H., and Z.L. edited and revised manuscript; L.A.J., K.A., T.N., W.B.H., and Z.L. approved final version of manuscript.
